# A WeChat-Based Mobile Platform for Perioperative Health Education for Gastrointestinal Surgery

**DOI:** 10.1155/2021/6566981

**Published:** 2021-11-25

**Authors:** Ying Li, Xiao-hui Cheng, Wen-ting Xu, Li-ping Tan, Xiao-jun Gou

**Affiliations:** ^1^The Second Affiliated Hospital of Soochow University, Suzhou, Jiangsu 215004, China; ^2^Baoshan District Hospital of Integrated Traditional Chinese and Western Medicine of Shanghai, Shanghai 201999, China

## Abstract

Appropriately instructing and guiding patients before and after surgery is essential for their successful recovery. In recent years, however, the development of the enhanced recovery after surgery (ERAS) protocol has restricted the opportunity for healthcare professionals to spend time with their patients before and after surgery because of efficiency-driven, shortened hospital stay. Here, we embedded health education information of the perioperative period for gastrointestinal surgery on a WeChat-based mobile platform and evaluated the platform through medical staff evaluation, patient volunteer evaluation, and quantitative grading rubric. Clinicians and nurses believed that the mobile platform was attractively designed and easy to navigate, valuable, and adequate for patient health education. The content of health education was embedded into the WeChat-based mobile platform, thereby allowing patients and caregivers to access information at their own pace and enable repeat reading.

## 1. Introduction

Although enhanced recovery after surgery (ERAS) has rapidly developed worldwide, a survey conducted showed that many patients were not adequately prepared for surgery and did not have the relative knowledge of certain diseases, which made them anxious [1]. These changes in clinical practice thus negatively affected the provision of information and postoperative instructions tailored to individual patients. Consequently, preoperative and postoperative health behavior compliances have decreased [2]. Hence, it is necessary to gain more health education knowledge in a shorter time [3]. Patients will be unable to take full advantage of minimally invasive surgery until they receive timely, accurate, and easily accessible health information in the perioperative period. The term “mHealth” is defined as the use of mobile devices for medical purposes or to support overall health and well-being [4, 5]. The ubiquity of mobile technology offers tremendous opportunities for the healthcare industry to address one of the most pressing global challenges, making healthcare more accessible, faster, better, and cheaper [6]. WeChat conformed to the trend of digitalization of information and integrated different modes of information that were acceptable to almost all audiences, such as pictures, texts, audio, and video. Users viewed the information flexibly without time and geographical constraints. The first research report of the use of WeChat platform showed that WeChat covered more than 94% of China's smartphone users, with monthly active users reaching 1,082.5 million. On average, 570 million users (93%) in China's first-tier cities logged on to WeChat every day [7]. A total of 1,636 questionnaires from WeChat customers were collected from 32 provinces. Nearly one-third of the users frequently received and read health information through WeChat [8]. Here, we embedded health education information of the perioperative period for gastrointestinal surgery on a WeChat-based mobile platform, as described.

## 2. Design of Perioperative Health Education on a Mobile Platform

**Figure 1:**
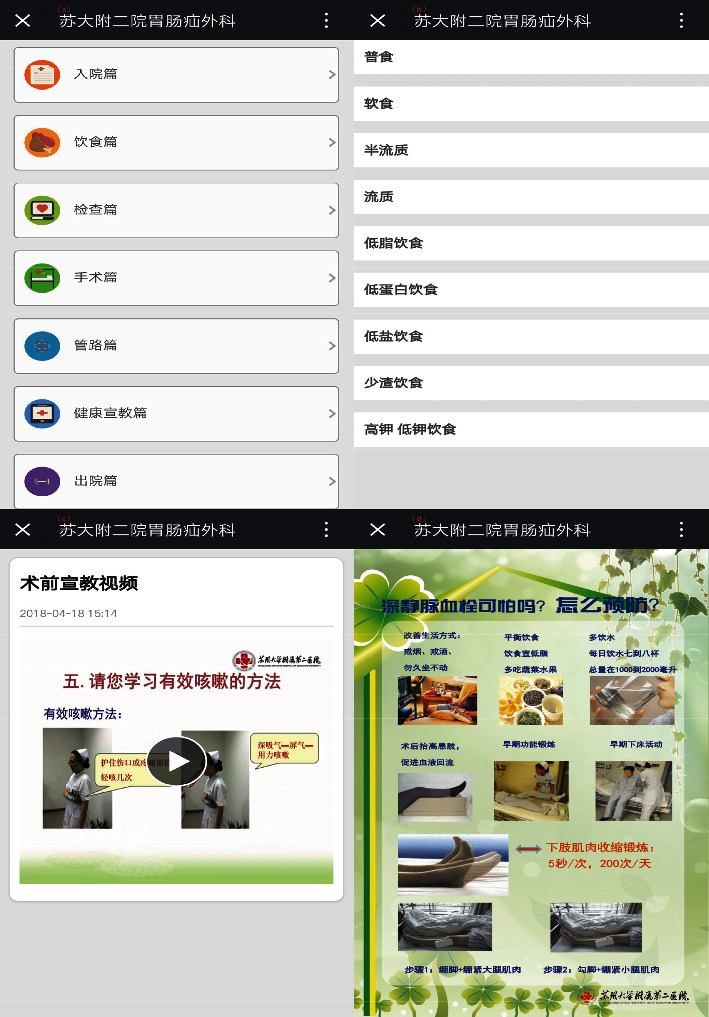
The interface of the perioperative health education mobile platform (originally in Chinese). (a) Seven components of the mobile platform, (b) the diet component, (c) video for effective coughing, and (d) deep vein thrombosis prevention education.

Our team developed The Perioperative Health Education Mobile Platform with the technological support of a specialized information technology company. The platform included the following seven components: guidance for new hospital patients, diet, examination, preoperative and postoperative guidance, pipeline care, educational material, and discharge guidance (Figure 1). Each component is described in detail later. Seven list buttons were placed linearly for navigation within the platform. This potentially encouraged patients to further browse through other health education contents to meet more of their needs (Figure 1). Each of the modules used media elements in various forms.

The guidance for new hospital patients was presented as a video. According to Mayer's Cognitive Theory of Multimedia Learning [9], when words and pictures were presented together, learners had the opportunity to form a mental model of speech and images and establish a connection between the two, which was better than words alone.

In the diet component, considering the characteristics of dietary transition in patients undergoing gastrointestinal surgery, we divided the diet into general food, soft food, semiliquid diet, liquid diet, low-fat diet, low-protein diet, low-salt diet, less slag diet, low-potassium diet, and high-potassium diet. The dietary principles were stated in each diet category. A survey [10] revealed that many patients knew what they could not eat during their hospital stay, but what puzzled them was the food suitable for eating. Therefore, in each food category, we not only listed foods that were restricted but also foods that were suitable for eating (Figure 1).

We listed the location, time, whether the patient needed an appointment, process, and precautions to be taken for each surgical examination. In addition, there was a detailed description of gastrointestinal disease-related tests such as methods of bowel preparation in colonoscopy. For some long-term examinations such as magnetic resonance imaging, there was a “long queue time” reminder, so that patients and their families will be psychologically prepared in advance and the medical staff could reasonably arrange the resources, thus reducing the anxiety of patients and their families and avoiding conflicts between medical staff and patients.

The preoperative preparation was presented as video and text materials. Items that could not be brought into the operating room were depicted as pictures. In addition to the routine preoperative preventive measures, an explanation was given on following certain Chinese cultural characteristics, such as reminding to remove the amulet. High-quality counseling of patients improves postoperative recovery [11] and thus shortens hospital stay, as recovery after minimally invasive surgery is slower than expected [12, 13]. Providing optimum guidance and information will gradually promote lifestyle changes and self-health skills. For example, many patients did not change their old concepts and fear to move after surgery or even lie down; this coupled with the various intubations for monitoring, wound pain, and other factors that hindered the early postoperative activities of patients. We detailed the preparation, method, and timing of each activity step as a flowchart. This could not only help the nursing staff but more importantly, it could encourage the patients to participate in self-management and promote their postoperative rehabilitation.

**Figure 2:**
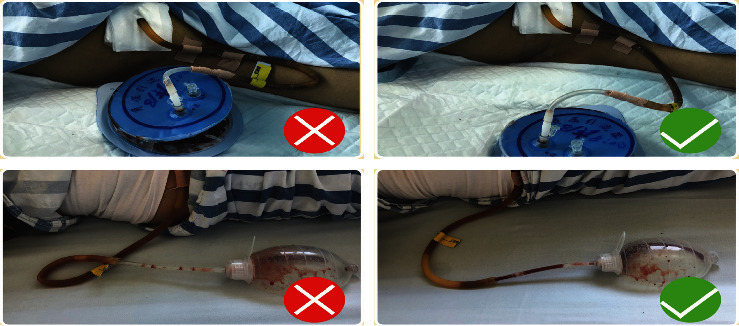
Pictures of drainage tube compression and distortion.

After gastrointestinal surgery, the patients were fitted with various intubations such as abdominal drainage tube, fistula, nasogastric tube, wound drainage tube, and central venous catheter (CVC). The most commonly used intubation procedure in our department was the abdominal drainage tube. Health education was provided as comics in which various characters were played by doctors, nurses, and patients. It was summarized as “one two three memory mouth:” one fixed, two smooth, three negative pressure, which was convenient for patients to remember. In addition, pictures of drainage tube compression and distortion that often occur during patient activities were presented to depict clinical real-life scenarios and to inform the patients about the emergencies that should be notified to the medical staff (Figure 2).

Education should provide tactical skills (such as how to follow a functional exercise strategy) and situational skills (such as how to decide whether to take painkillers). Adequate knowledge is necessary for patient self-care management. To meet this need, a WeChat-based platform embedded a health education module that provided links to relevant information (e.g., pain management and deep vein thrombosis prevention). In the Health Mission module, we used problem-based learning, a patient-centered approach that promoted patient involvement in health management when patients experienced negative health effects [14]. In this module, for example, the nurse used a synchronized form of video, audio, and text to show the effective method of coughing. When words were presented in an auditory form, people could process them through their brain auditory/language channel, and the brain visual/image channel could only be used to process images. This did not place an excessive load on brain processing, thereby enabling the patient to understand the information more effectively (Figure 1). The steps of lower limb muscle exercise to prevent deep vein thrombosis were presented as patient role play, and key points were attached to each corresponding picture. Sweller advocated minimizing external cognitive load due to the organization and presentation of learning materials. Learners should be provided with “constructed” good learning materials, where graphics have been integrated, redundant information has been eliminated, and so on [15] (Figure 1).

The discharge guidance components listed the materials that needed to be prepared at the time of discharge, the procedures for discharge, drug reminders, and departmental contact information. In addition, discharge guidance was modified according to different disease types and different types of surgery. For example, discharge guidance differed for patients with gastric cancer, colon surgery, rectal surgery, and chemotherapy.

**Figure 3:**
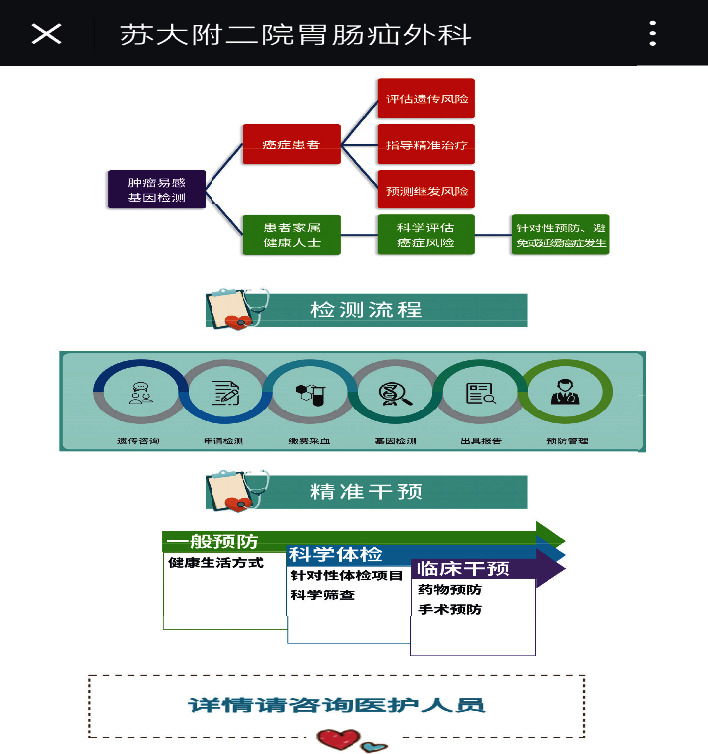
The gastrointestinal tumor gene screening module.

In addition, our department of gastroenterology successfully introduced a multidisciplinary diagnosis and treatment team for gastrointestinal cancer at the Japan Cancer Institute Hospital in 2018, which was the first international clinical team in Suzhou. Taking advantage of this opportunity, we added the gastrointestinal tumor gene screening module in the mobile platform (Figure 3). According to the characteristics of patients admitted to our department of gastroenterology, the ostomy care home and pipeline care were added in the module.

In addition, we used appropriate techniques to compress audio, images, and video to make the file stream play easily and quickly. To increase consistency, navigation was provided on the left side of each page, and the title was given at the top of each page to provide a valid user experience. We made changes to the design and content on the basis of feedback received from tests in both patients and healthcare professionals. Furthermore, we provided a QR code to enable users to conveniently and freely access the App. The color scheme and font type and size of the text were chosen by considering age-related changes in older adults [16]. There was no need to download the App specifically, which reduced development costs.

## 3. Team Division

Nurses with master's degree in nursing and working in the gastrointestinal department were responsible for checking the literature, guidelines, and other information pertaining to gastrointestinal disorders and integrating the knowledge gained into the health education content. General surgeons, nutritionist dietitians, anesthesiologists, and enterostomal therapists collaborated to review and supplement content. Information technology technicians skilled in developing user interface were responsible for the compatibility between the content and the mobile platform, for maintaining the network link, and for implementing the design concept on the layout of the mobile platform. In addition, a liaison was assigned to coordinate the work of general surgeons, dieticians, anesthesiologists, enterostomal therapists, nurses, and information technology technicians.

## 4. Evaluation of Perioperative Health Education Mobile Platform

### 4.1. Medical Staff Evaluation

Before the mobile platform generated the QR code link, four clinicians (two in general surgery, one in nutrition department, and one in the anesthesiology department) and four clinical nurses evaluated the content and design of the mobile platform. The doctors (*N* = 4) believed that the content of the platform was consistent with the current medical practice and clinical guidelines. The nurses (*N* = 4) considered that providing health education on mobile platforms can help to reduce repetitive task of giving the required health information. Both clinicians and nurses considered that the design of the mobile platform was attractive and easy to navigate, especially in the role of patient to demonstrate care operations that promote patient health issues.

### 4.2. Patient Volunteer Evaluation

Twenty patient volunteers were recruited after receiving a brief overview of the purpose and scope of this platform. Twelve participants were males (60%) and eight participants were females (40%). The mean age of the participants was 52 years and ranged from 45 to 72 years of age; 60% of the participants were 50 years or older. All the participants had undergone gastrointestinal surgery. There were differences in their level of education. Participants were required to use the mobile platform for up to 1 hour or until feedback could be given [17]. The liaisons actively collected the opinions of the participants during their use of the mobile platform. Once the users completed the navigation, they were asked to complete a questionnaire consisting of 9 questions. Four of these questions focused on topics related to whether the patient had obtained adequate health education information about perioperative care for gastrointestinal surgery and whether the user learned new information from the content provided. The remaining five questions assessed whether the platform was familiar and convenient to use and whether it was simple enough for them to learn and navigate. All the participants completed the study and used WeChat, and 70% of the participants used WeChat more than 10 times a day (Table 1).

### 4.3. Quantitative Grading Rubric

Our mobile platform was reviewed using a quantitative grading rubric developed by the researchers. In a prior evaluation, Handel [18] reviewed 35 health and wellness mobile apps on the basis of ease of use, reliability, quality, scope of information, and aesthetics. Aisha Masud [19] developed a quantitative rubric and graded 44 dermatology mobile apps with the primary focus on patient education. These criteria were modified and adapted for the purposes of the present study, and a 4-point scale was used for each criterion. The final criteria were (1) educational objectives, (2) content, (3) accuracy, (4) design, and (5) conflicts of interest. The quantified grading rubric is given in Table 2. The total score was 5–20 points, and the evaluation score was as follows: 5–10 points, useless and may even be harmful to the patient; 11–15 points, could be used for patient health education, but there are some limitations; and 16–20 points, valuable and adequate for patient health education. Ten patients, doctors, and nurses evaluated each of the abovementioned five dimensions.

Patients (*N* = 100), doctors (*N* = 50), and nurses (*N* = 50) evaluated the health education mobile platform. The evaluation scores for all were >15 points, which indicated that the mobile platform was valuable and adequate for patient health education (Table 3).

## 5. Discussion

Perioperative patients are often “fragile.” Health education is an important part of perioperative care. Perioperative health education content of gastrointestinal surgery involves the entire process of preoperative preparation, postoperative pain management, tube nursing, complication identification, and stoma care. The contents were complicated; moreover, some patients had difficulty in reading due to factors such as age, health condition, and cultural level. Furthermore, written words could not be preserved for a long time, which led to noncompliance of perioperative care. In traditional health education, patients usually receive brief verbal instructions or preprepared task papers. However, nurses are often in a hurry when performing these tasks. The health education mobile platform could solve the problems encountered in the traditional approach. Providing adequate and convenient perioperative care could foster a trusting relationship between patients and medical staff.

Evaluation tests helped to understand patient preferences and improve the perioperative health education content of gastrointestinal diseases embedded in mobile platforms. In the present study, the evaluation scores of patients, doctors, and nurses were >15 points. The health education mobile platform was valuable for patients with gastrointestinal diseases who are undergoing surgery. In the dimension of educational goals, patients scored 2.9 points, which was lower than that of other dimensions. This may be because of the patients' condition and the degree of understanding, which led to different educational needs. Some patients mentioned that although they understood the corresponding content, they still hoped to receive personal explanation from the medical staff. The patients and the medical staff involved in the survey indicated that the technical difficulties associated with the use of the mobile platform were very small. Our education module provided simpler and lesser text and required only the scanning of the QR code; this was simpler than using mobile devices to send a website to a patient for the continuation of care, which required the patient to log in to the website after registration to read the relevant information for learning [20]. The major advantage was immediate access to the health education content, which allowed to learn in real time. Furthermore, 90% of the participants of the beta test indicated that they were familiar with the scanning QR code function of WeChat. A 78-year-old patient only used WeChat for checking information and found that it was easy to manage under the guidance of the family. According to the 2018 WeChat data report, 63 million users over the age of 55 years were active users every month and paid attention to health and wellness. Inexperience with smartphones and apps will be less of an issue as the older adult population continues to adapt to the technology [21]. Therefore, the health education mobile platform can promote family members to participate in the health management of patients, which will enable the patients to learn and review health information at any time. As 20% of the participants were undergoing secondary surgery, the remaining 80% of the participants reported learning new information.

The rising trend in the usage of smartphones and mobile tablets in the general population highlighted the need for multimedia to facilitate the delivery of surgical and medical information. Highly accessible health education material on mobile platform would also be effective in delivering information to patients. It is therefore essential for medical staff to provide validated patient education materials by leveraging smart device technology. A survey study in a comparable population showed that 78% would prefer to use e-health in perioperative care [22].

The first research report of the WeChat-based platform showed that more than 90% of the users used WeChat every day, and half of the users used WeChat for more than 1 hour every day [7]. Mounting evidence showed that symptom assessment using technology assisted older adults to gauge their health status and manage chronic illnesses [8, 23]. A total of 15,310 participants were enrolled in a weight loss intervention campaign, among which 77.35% were willing to use WeChat for monitoring weight loss; this finding was consistent with a study in which WeChat health education program received high levels of satisfaction from the participants. Another study showed that the more the number of active male WeChat users, the more was the weight they lost [24].

The paucity of studies on this topic was remarkable considering the increasing popularity of minimally invasive techniques performed in day-care settings in this area and the associated lack of postoperative guidance in the postoperative course, which created an urgent need for e-health solutions. In a systematic review on the effects of e-health in perioperative care, 11 studies were identified in which educational e-health interventions were evaluated [25]. However, only one study was performed in patients undergoing abdominal surgery [26]. As indicated in previous research, there was a need to improve and refine the content on WeChat, such as developing various materials to attract interest of the target population. Future research should focus on how to improve adherence to the WeChat Perioperative Health Education interventions for gastrointestinal surgery.

Embedding the content of health education into the WeChat-based mobile platform allowed patients and caregivers to access information at their own pace and enables repeat reading. Compared to traditional video education, the mobile platform can be accessed on any smart device, which was conducive to permanent storage and does not occupy memory. Perioperative health education on mobile platforms combined images, animations, and video content based on patient perspectives to make health knowledge more intuitive and unobtrusive, independent of space and economic conditions, and more suitable for low understanding patients and visual learners in today's society.

Therefore, in the future, there should be greater focus on how to improve adherence to health education interventions, and further studies on the usage of the mobile platform for a longer period of time are necessary.

## 6. Conclusion

Embedding health education on mobile platforms not only offered health guidance to individuals but also provided them with basic, preventive treatments that were accessible any time. Having the correct information accessible on a mobile phone was reported as a health coach or companion that promoted persistent engagement to improve health outcomes by all patients. Next, we plan to apply the use of the mobile platform to a wider range of patients to test for improved clinical outcomes and to demonstrate continued benefits. An increasing number of people are beginning to use mobile devices such as smartphones to understand medical information, and mobile phones are kept handy throughout the day to receive constant updates on health education and behavioral interventions. Therefore, future research studies should focus on using mobile platforms to improve health. This improvement in recovery time, together with a shortened hospital stay, an increase in surgical volume, and the growing popularity of technology in this area, suggested that a personalized, perioperative, e-health program should be considered for implementation in standard perioperative care. With the widespread use of WeChat and the large number of active users, WeChat may be a convenient, cost-effective medium to improve adherence to perioperative care behaviors in China.

## Figures and Tables

**Table 1:** Summary of beta testing results.

Content	Number	Percentage (%)
Mobile platform is educational	20	100
Learned new knowledge that you did not know before	16	80
Perioperative care can be learned	20	100
Associated with gastrointestinal surgery	20	100
Mobile platform navigation is simple	20	100
The form of the video is helpful	20	100
The form of patient role playing is helpful	20	100
The form of the picture combination is helpful	20	100
Familiar with scan QR code function using WeChat	18	90

**Table 2:** Quantitative grading rubric used for the review of the mobile platform.

Category	1	2	3	4
Educational objectives	Mobile platform does not fulfills the focus and educational objectives of its description.	Mobile platform minimally fulfills the focus and educational objectives of its description.	Mobile platform mostly fulfills the focus and educational objectives of its description.	Mobile platform completely fulfills the focus and educational objectives of its description.
Content	Mobile platform has major gaps in information; it is disorganized and confusing.	Mobile platform has gaps in information, and the content is disorganized.	Mobile platform has minor gaps in information relayed or is disorganized.	Information provided in the mobile platform is complete, comprehensive, and logical.
Accuracy	Mobile platform presents factually incorrect information that detracts from the educational objectives.	Mobile platform has minor errors that do not detract from the educational objectives.	Mobile platform has no factual errors; however, it does not provide resources.	Mobile platform provides evidence based, factually correct information.
Design	Design of the mobile platform is difficult to use and obtrusive to the relaying of information to the user.	Mobile platform has some issues with design that may have minor interference with relaying of information to the user.	Mobile platform has a design, interface, and mode of navigation that are understandable and do not hinder the relaying of information to the user.	Mobile platform is easy to use and well designed with an interface and mode of navigation that is understandable and enhance the user experience.
Conflicts of interest	Mobile platform has obvious conflicts of interest resulting in selective, biased, or misleading information.	Mobile platform was made with some conflicts of interest, however, it presents information in a mostly unbiased and objective way.	Mobile platform is created with some sort of monetary incentive; however, it relays unbiased, factually correct information.	Mobile platform was created with no conflicts of interest or monetary incentive and has the sole purpose of relaying educational information.

**Table 3:** Results of quantitative grading rubric evaluation.

Content	Patient	Doctor	Nurse
Educational objectives	2.9	3.2	3.6
Content	3.1	3.6	3.5
Accuracy	3.5	3.6	3.7
Design	3.7	3.7	3.5
Conflicts of interest	3.8	3.7	3.8
The average score	17.0	17.8	18.1

## Data Availability

The data used to support the findings of this study are available from the corresponding author upon request.
